# LIFU Alleviates Neuropathic Pain by Improving the KCC_2_ Expression and Inhibiting the CaMKIV–KCC_2_ Pathway in the L4–L5 Section of the Spinal Cord

**DOI:** 10.1155/2021/6659668

**Published:** 2021-04-13

**Authors:** Ye-Hui Liao, Bin Wang, Mo-Xian Chen, Yao Liu, Li-Juan Ao

**Affiliations:** School of Rehabilitation, Kunming Medical University, Kunming, 650500 Yunnan Province, China

## Abstract

Effective treatment remains lacking for neuropathic pain (NP), a type of intractable pain. Low-intensity focused ultrasound (LIFU), a noninvasive, cutting-edge neuromodulation technique, can effectively enhance inhibition of the central nervous system (CNS) and reduce neuronal excitability. We investigated the effect of LIFU on NP and on the expression of potassium chloride cotransporter 2 (KCC_2_) in the spinal cords of rats with peripheral nerve injury (PNI) in the lumbar 4–lumbar 5 (L4–L5) section. In this study, rats received PNI surgery on their right lower legs followed by LIFU stimulation of the L4–L5 section of the spinal cord for 4 weeks, starting 3 days after surgery. We used the 50% paw withdraw threshold (PWT_50_) to evaluate mechanical allodynia. Western blotting (WB) and immunofluorescence (IF) were used to calculate the expression of phosphorylated extracellular signal–regulated kinase 1/2 (p-ERK1/2), calcium/calmodulin-dependent protein kinase type IV (CaMKIV), phosphorylated cyclic adenosine monophosphate response element-binding protein (p-CREB), and KCC_2_ in the L4–L5 portion of the spinal cord after the last behavioral tests. We found that PWT_50_ decreased (*P* < 0.05) 3 days post-PNI surgery in the LIFU^−^ and LIFU^+^ groups and increased (*P* < 0.05) after 4 weeks of LIFU stimulation. The expression of p-CREB and CaMKIV decreased (*P* < 0.05) and that of KCC_2_ increased (*P* < 0.05) after 4 weeks of LIFU stimulation, but that of p-ERK1/2 (*P* > 0.05) was unaffected. Our study showed that LIFU could effectively alleviate NP behavior in rats with PNI by increasing the expression of KCC_2_ on spinal dorsal corner neurons. A possible explanation is that LIFU could inhibit the activation of the CaMKIV–KCC_2_ pathway.

## 1. Introduction

Neuropathic pain (NP) is defined as pain originating from primary lesions and dysfunction of the somatosensory system, either at the peripheral or central level [1]. Many studies have been conducted on this type of pain, and some progress has been made, but many challenges remain in the clinical treatment of NP [2]. The main clinical manifestations include spontaneous pain, persistent (or paroxysmal) pain, induced pain, paresthesia, numbness, and tingling [2, 3]. As a refractory and chronic pain that can manifest in various ways, including as chronic low back pain or sciatica, NP is a severe problem. It affects 6.9%–10% of the population worldwide and seriously diminishes patient's quality of life [2], increasing the economic burden on the patient's family and on society [4–6].

The etiology and mechanism of NP are complicated and unclear. Recently, an increasing amount of evidence has shown that downregulation of potassium chloride cotransporter 2 (KCC_2_) in the spinal cord plays an important role in NP. KCC_2_ is an ion transporter protein present in mature neurons of the central nervous system (CNS) and can remove Cl^−^ from the cytoplasm to the extracellular space [7]. After peripheral nerve injury (PNI), the expression of KCC_2_ on the neuronal membrane is downregulated, and the concentration of Cl^−^ ([Cl^−^]_i_) in nerve cells is upregulated, thereby reducing the inhibitory effect of the neurotransmitter *γ*-aminobutyric acid (GABA) [8–10]. GABA, the main inhibitory neurotransmitter in the mature CNS [11], can bind to the GABA receptor (GABA-R) to promote depolarization of the postsynaptic nerve membrane and mediate hyperpolarization and activity of the neuron [12]. Dysfunction of GABA-R eventually reduces inhibition of the spinal cord, leading to hyperexcitability of primary afferent neurons and activation by low-threshold mechanical sensory input. Simultaneously, the primary afferent neurons respond only to high-threshold (nociceptive) inputs under normal circumstances, thereby causing mechanical allodynia and NP [13, 14]. NP has been successfully induced in rats by injecting microribonucleic acid (miRNA), which interferes with the transcription of KCC_2_, or a KCC_2_ inhibitor [15, 16]. All results suggest that downregulation of KCC_2_ after PNI plays an essential role in the development of NP [17]. Many studies have found that increasing the expression of KCC_2_ significantly relieves NP behavior [9, 18–20]. Therefore, learning how to increase the expression of KCC_2_ following PNI has great potential value for treating NP.

**Figure 1:**
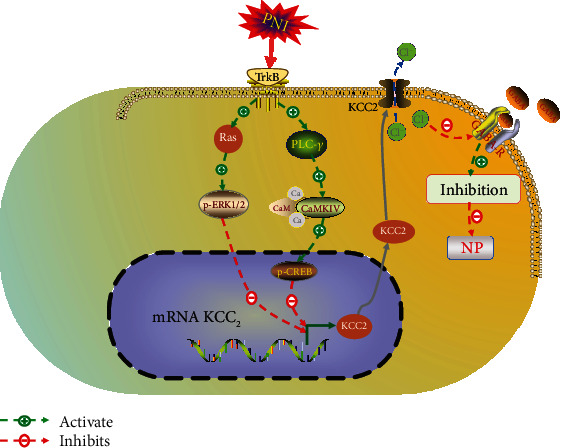
Outline of the current view on the roles of the p-ERK–KCC_2_ and CaMKIV–KCC_2_ signaling pathways after PNI in the induction of NP. Under normal conditions, KCC_2_ extrudes intracellular Cl^−^ ions from the cell and maintains the inhibitory effect mediated by GABA receptor. PNI activates TrkB and then obstructs the translation of KCC_2_ through the p-ERK–KCC_2_ and CaMKIV–KCC_2_ signaling pathways.

After PNI, nociceptive stimulation leads to downregulation of the KCC_2_ expression through a series of intracellular cascades in the brain-derived neurotrophic factor- (BDNF-) tropomyosin receptor kinase B (TrkB) pathway [21, 22]. Calcium/calmodulin-dependent protein kinase type IV (CaMKIV), phosphorylated cyclic adenosine monophosphate response element-binding protein (p-CREB), and phosphorylated extracellular signal–regulated kinase 1/2 (p-ERK1/2) play essential roles in activation of the BDNF–TrkB pathway cascades and downregulation of the KCC_2_ expression [10, 23, 24]. Rivera confirmed in transgenic mice that activation of the TrkB receptor by BDNF further inhibited the expression of KCC_2_ at the transcriptional level via the intracellular phosphoinositide phospholipase C gamma (PLC*γ*)⟶Ca^2+^⟶CaMKIV⟶p − CREB cascade [23]. Recently, studies also have found that nociception such as PNI or inflammation can activate the ERK–mitogen-activated protein kinase (MAPK) pathway in the spinal dorsal horn, upregulate the intracellular p-ERK1/2 expression via a Ras⟶p-ERK1/2 cascade reaction, and inhibit the expression of KCC_2_ at the transcriptional level, ultimately leading to NP [23, 25, 26]. Therefore, the CaMKIV–KCC_2_ or p-ERK1/2–KCC_2_ pathway plays a vital role in NP pathogenesis after PNI (Figure 1).

At present, due to its complicated mechanism, there is still no satisfactory treatment for NP [27, 28]. At the clinical level, conventional painkillers such as tricyclic antidepressants, anticonvulsants, nonsteroidal anti-inflammatory drugs (NSAIDs), antiepileptic drugs, and weak and strong opioids are often used for symptomatic relief, but with poor efficacy and many side effects [28–30]. Therefore, finding a suitable rehabilitation method for NP would have great clinical significance. As a form of noninvasive neuromodulation, low-intensity focused ultrasound (LIFU) has been confirmed safe for modulating brain activity in patients and animals with seizures [31], Alzheimer's disease and dementia [32], traumatic brain injury (TBI) [33], and depression [34]. LIFU's neuromodulatory mechanism includes mechanical, thermal, and cavitation effects [35]. The mechanical effect of LIFU plays an important role in neuromodulation, and its mechanism might be that acoustic radiation forces the bimolecular structure of the cell membrane to stretch through mechanical vibration, thereby interfering with the mechanically sensitive ion channels on the cell membrane and producing the corresponding biological effect [36, 37]. Interestingly, King et al. applied LIFU to the CNS in epileptic rats and found that it could inhibit abnormal epileptic discharge by activating GABAergic neurons in the CNS [38].

However, whether spinal cord stimulation with LIFU can enhance the inhibitory effect and alleviate NP is still unclear. In this study, we loosely ligated the right tibial nerve and common peroneal nerve in rats to create a PNI model. After LIFU stimulation of the L4–L5 spinal cord section, we used the 50% paw withdraw threshold (PWT_50_) to evaluate the rats' mechanical stimulation threshold; WB and IF were used to detect the expression changes of p-ERK1/2, CaMKIV, p-CREB, and KCC_2_ in the lumbar spinal cord.

## 2. Materials and Methods

### 2.1. Animals

We acquired a total of 40 healthy male Sprague-Dawley (SD) rats (weight, 220–300 g) from Kunming Laboratory Animal Center (Kunming, China) for use in the experiment. All rats were housed at 25°C ± 2°C on a 12 h reverse light/dark cycle in separate cages (5 rats per cage) and had free access to food and water. All animal protocols were approved by the Animal Ethics Committee of Kunming Medical University (No. KMMU2020352).

### 2.2. Grouping and Experimental Design

After 1 week of adaptation, all rats were randomly divided into four groups (10 per group): normal group, rats that received neither surgery nor treatment; sham group, rats in which nerves were exposed according to the PNI surgical method but not ligated; and LIFU^−^ group and LIFU^+^ group, rats that received PNI surgery and LIFU stimulation in parallel, except that the ultrasound (US) amplifier was always turned off during treatment in the LIFU^−^ group.

### 2.3. PNI Model of NP

We developed the PNI model using the selective nerve injury (SNI) method in strict accordance with the literature [39]. Rats were anesthetized by intraperitoneal (i.p.) injection of 1% sodium pentobarbital (40 mg/kg). We shaved the fur at the right knee joint's proximal end and made a 1 cm incision. The muscle was separated bluntly, layer by layer, followed by exposure of the three branches of the right sciatic nerve: the tibial nerve, the common peroneal nerve, and the sural nerve. The common peroneal and tibial nerves were loosely ligated with 4-0 silk in three places at 1 mm intervals. We carefully performed manipulations during ligation to avoid injuring the sural nerve. The branches of the right sciatic nerve were exposed but not ligated in sham group rats.

### 2.4. LIFU Stimulation of the L4–L5 Spinal Cord Section

**Figure 2:**
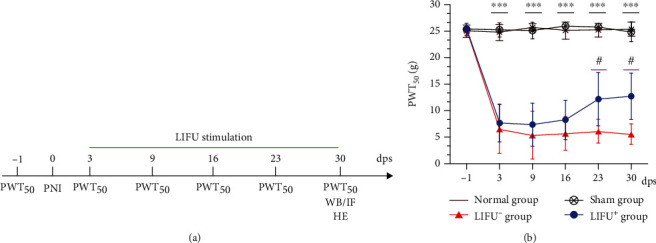
(a) Timeline of experimental protocol. Dps: days postsurgery. (b) Therapeutic effects of LIFU stimulation on NP in PNI rats. Mechanical allodynia (PWT_50_) was significantly decreased in the LIFU^−^ and LIFU^+^ groups 3 days after PNI surgery compared with the normal and sham groups. After 3 weeks of LIFU treatment, PWT_50_ increased compared with the LIFU^−^ group. Each symbol represents the mean ± SEM; ^∗∗∗^*P* < 0.001 against the LIFU^−^ and LIFU^+^ groups, #*P* < 0.05 against the LIFU^−^ group. One-way ANOVA; *n* = 10 per group.

LIFU stimulation was started on the third day after PNI surgery during the time range of 09 : 00–15 : 00 (Figure 2(a)). After administering mild mixed anesthesia with isoflurane and sodium pentobarbital, we fixed the rats on a table and applied a depilatory cream to remove the fur on their backs, exposing the L4–L5 spinal segment. The transducer was fixed on this segment, and the skin was covered, and the transducer gaps filled with an ultrasonic coupling agent (Aquasonic; Parker Laboratories, Fairfield, NJ, USA) without bubbles. Parameters were as follows: sine pulse wave frequency, 4 MHz; duty cycle (DC), 20%; pulse repetition frequency (PRF), 0.8 KHz; irradiation intensity, 0.65 MPa; and treatment duration, 20 min/d for 4 weeks. We calibrated the beam's irradiation intensity using a hydrophone (HNR 0500; Onda, Sunnyvale, CA, USA).

### 2.5. Tissue Preparation

After the last LIFU treatment and behavioral test, rats were sacrificed via overdose of 1% sodium pentobarbital (40 mg/kg), and tissues were harvested for WB (*n* = 5) and IF (*n* = 5) staining analysis. For WB, we rapidly collected L4–L5 spinal cord section tissues and stored them at −80°C until use. For IF, rats were perfused with 200 ml prechilled 0.9% saline (4°C) and then 150 ml prechilled 0.1 M phosphate buffer (pH 7.4) containing 4% paraformaldehyde (4°C). We harvested the L4–L5 spinal cord section, fixed it in 4% paraformaldehyde overnight at 4°C, and separately dehydrated the slices one by one for 24 h using 20 and 30% sucrose 0.9% saline solution. After being embedded with optimal cutting temperature (OCT) compound, the transverse section slice (8–12 *μ*m thick) of the spinal cord was used for IF or hematoxylin and eosin (H&E) staining.

### 2.6. Assessment of LIFU Safety

We performed H&E staining to assess the safety of LIFU for the spinal cord. Sections were prepared according to the following procedures: fixation for 30 s, washing in water for 5 min, staining with hematoxylin solution for 5 min, dipping in 1% acid ethanol five times, staining with eosin solution for 2 min, dipping in graded alcohol (from a high to a low concentration) for 5 min per grade, washing in water for 15 min, dehydration with graded (from a low to a high concentration) alcohol, clearing with xylene, and mounting in resin. We used a digital microscope to observe the results of H&E staining.

### 2.7. Measurement of Mechanical Allodynia

Behavioral tests were performed in a controlled environment by investigators who were blinded to animal treatments. Each rat was separately placed in a metallic mesh cage (20 × 20 × 15 cm^3^) and allowed to adapt to the environment for 20 min before the test. We used the up-and-down method to test PWT_50_ as described in the literature [40]. A series of von Frey (VF) filaments (Stoelting, Wood Dale, IL, USA) with ascending degrees of stiffness (1.4, 2.0, 4, 6, 8, 10, 15, and 26 g) were used to irritate the ipsilateral plantar surface of the PNI paw. The first VF filament to be used was the 6 g filament, and appropriate force was used to bend each filament for 5 s. Licking, lifting, or removing the paw was considered a positive reaction. According to the negative or positive response, we applied a filament at a greater or lower degree of force. PWT_50_ was calculated as follows:

(1) $$\begin{array}{c} 50\%\text{g threshold}=\left(10^{\left[\text{x}f+\text{k}\delta \right]}\right)/10,000. \end{array}$$

The PWT_50_ test was performed presurgery for 1 day, and pre-LIFU stimulation was performed 1 day/week during the LIFU stimulation period.

### 2.8. Western Blotting (WB) Analysis

The spinal cord tissue (0.1 g) was dissected, homogenized via US, lysed with radioimmunoprecipitation assay (RIPA) buffer (RIPA: phenylmethylsulfonyl fluoride [PMSF] = 1 ml : 10 *μ*l) on ice for 30 min, and centrifuged at 12,000 r/min for 30 min at 4°C; then, we harvested the supernatants. Total protein concentration was quantified via a bicinchoninic acid (BCA) assay kit (Biomed, Beijing, China), and all samples were equalized to 30 *μ*g/10 *μ*l. Samples (total protein, 30 *μ*g) were resolved by 6%, 10%, and 12% sodium dodecyl sulfate polyacrylamide gel electrophoresis (SDS-PAGE) and transferred to polyvinylidene difluoride (PVDF) membranes (MilliporeSigma, Burlington, MA, USA). We blocked the membranes with 5% fat-free milk at room temperature (RT) for 2 h and then incubated them overnight with primary antibodies at 4°C with gentle shaking. These antibodies included monoclonal antibodies (mAbs) against CaMKIV (1: 2000; Abcam, Cambridge, UK), p-CREB (1: 1000; Cell Signaling Technology [CST], Danvers, MA, USA), p-ERK1/2 (1: 2000; CST), and KCC_2_ (1: 1000, CST), as well as glyceraldehyde 3-phosphate dehydrogenase (GAPDH; 1: 50,000; ABclonal Technology, Woburn, MA, USA) and *β*-actin (1: 2000; Santa Cruz Biotechnology, Dallas, TX, USA). The membranes were then incubated with a secondary antibody, horseradish peroxidase- (HRP-) labeled anti-rabbit/anti-mouse immunoglobulin G (IgG) HRP-linked antibody (1: 2000; CST), for 90 min at RT. Finally, we visualized and quantified protein bands using enhanced chemiluminescence (ECL; Tanon, Shanghai, China) and an ImageJ software (US National Institutes of Health [NIH], Bethesda, MD, USA). Protein was normalized based on *β*-actin or GAPDH concentrations.

### 2.9. Immunofluorescence (IF) Staining

For IF, each slice was washed in phosphate-buffered saline (PBS) for 10 min at RT and then incubated with 5% goat serum and 0.03% Triton X-100 in 0.1 M PBS for 2 h. Then, we incubated the slices in primary antibodies against KCC_2_ and p-CREB (respectively, 1 : 100 and 1 : 800; CST), as well as antibody against NeuN (1 : 1000, Abcam), at 4°C overnight. The secondary antibodies (anti-rabbit IgG [heavy + light (H + L) chain], F [ab′]2 fragment [Alexa Fluor 488 Conjugate]; anti-mouse IgG [H + L chain], F [ab′]2 fragment [Alexa Fluor 594 Conjugate]) were used for incubation at RT in the dark for 2 h. After three 10 min washes with PBS, we incubated the sections with 4′,6-diamidino-2-phenylindole (DAPI; Solarbio, Beijing, China). Images were captured under a fluorescence microscope (Olympus Corp., Tokyo, Japan). We used ImageJ software (US National Institutes of Health [NIH], Bethesda, MD, USA) to quantify the density of positive regions.

### 2.10. Statistical Analyses

Data are presented as mean ± standard deviation (SD). We used SPSS version 23.0 (IBM Corp., Armonk, NY, USA) for all statistical analyses. GraphPad Prism software version 8.0 (GraphPad Software, Inc., San Diego, CA, USA) was used to generate graphs. After verifying that all data were normally distributed, we used one-way analysis of variance (ANOVA) to analyze PWT_50_, WB, and IF data. *P* < 0.05 was considered statistically significant.

## 3. Results

### 3.1. H&E Staining of the L4–L5 Spinal Cord Section Was Used to Observe the Safety of LIFU Stimulation

**Figure 3:**
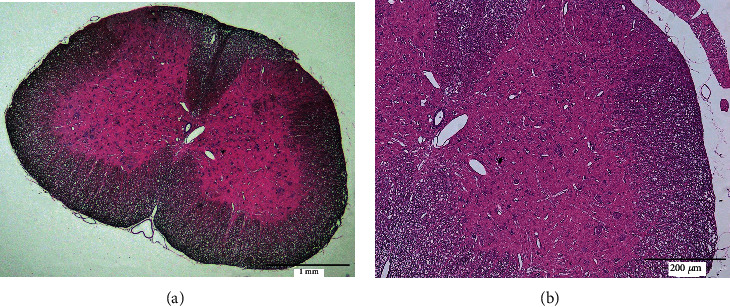
H&E staining showed that LIFU was safe for stimulating the spinal cord ((a) ×40, scale bar = 1 mm; (b) ×100, scale bar = 200 *μ*m) L4–L5 section of the spinal cord, showing no edema, hemorrhage, or cell necrosis.

We saw no swelling or nuclear fragmentation of neurons, neutrophil infiltration, or bleeding under cross-sectional magnification (Figure 3(a), ×40; Figure 3(b), ×100) of this spinal cord section.

### 3.2. LIFU Alleviated Mechanical Allodynia in PNI Model Rats

As shown in Figure 2(a), we used PWT_50_ to assess the effect of LIFU stimulation on PNI rats at different times. One day before LIFU stimulation, PWT_50_ had significantly decreased from 25.3 ± 1.2 g (LIFU^−^ group) and 25.4 ± 1.1 g (LIFU^+^ group) to 6.6 ± 4.6 g and 7.7 ± 3.6 g, respectively (*P* <0.05), but there was no statistically significant difference between the two groups (*P* > 0.05). After LIFU stimulation, PWT_50_ gradually increased, eventually becoming higher in the LIFU^+^ group (12.1 ± 5.0 g) than in the LIFU^−^ group (6.1 ± 2.2 g) after 3 weeks of LIFU stimulation (*P* < 0.05) and remaining stable to the end of LIFU stimulation. However, it was still lower in the normal and sham operation groups (*P* < 0.05), which there was no significant difference (*P* > 0.05; Figure 2(b)).

### 3.3. LIFU Stimulation Increased the KCC_2_ Expression in the L4–L5 Spinal Cord Section

**Figure 4:**
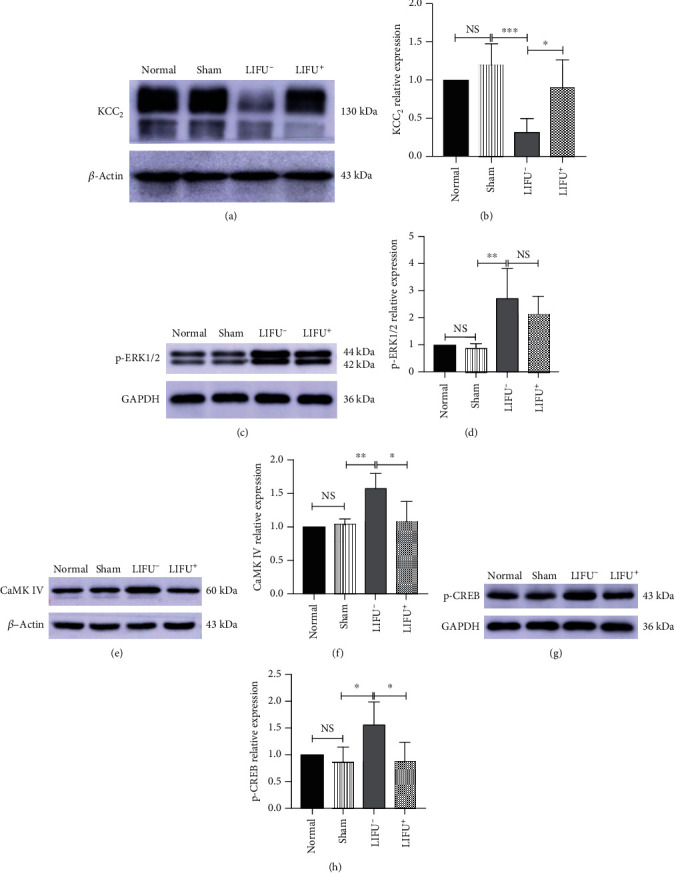
WB analysis of KCC_2_ (a, b), p-ERK1/2 (c, d), CaMKIV (e, f), and p-CREB (g, h) expression in the L4–L5 section of the spinal cord in different groups at 4 weeks post-LIFU treatment. Values, normalized to *β*-actin, or GAPDH. Each symbol represents the mean ± SEM; ^∗^*P* < 0.05, ^∗∗^*P* < 0.01, ^∗∗∗^*P* < 0.001. One-way ANOVA; *n* = 5 rats per assay.

**Figure 5:**
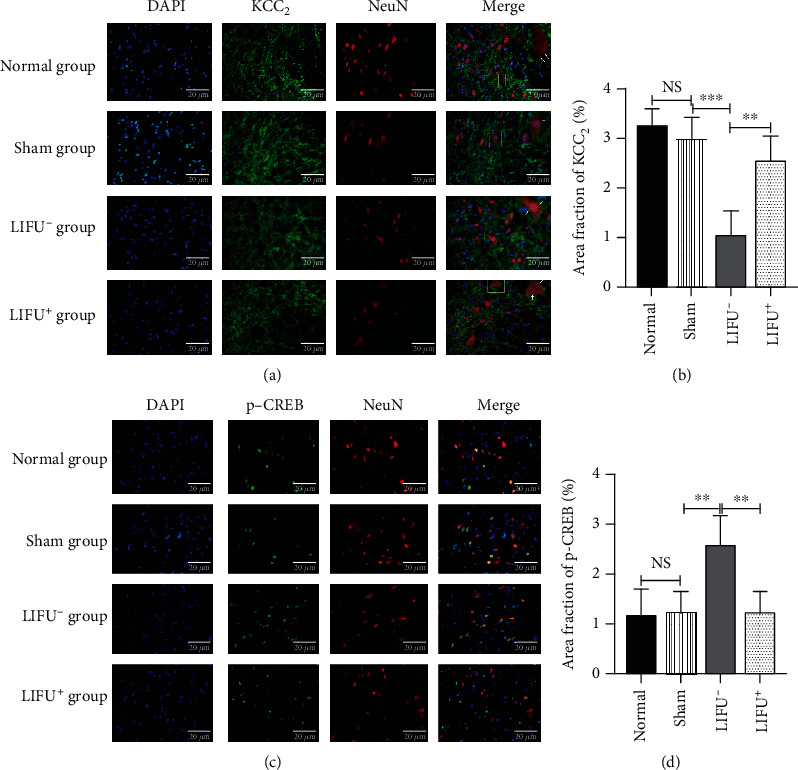
Expression of KCC_2_ (a) p-CREB (c) in the spinal cords of rats in different groups (IF, ×400). Scale bar = 20 *μ*m. Intensities of KCC_2_ (b) and p-CREB (d) IF in the spinal cords of rats in different groups after 4 weeks of LIFU treatment. Each symbol represents the mean ± SEM; ^∗^*P* < 0.05, ^∗∗^*P* < 0.01, ^∗∗∗^*P* < 0.001. One-way ANOVA; *n* = 5 rats per assay.

After 4 weeks of LIFU stimulation, rats were sacrificed, and the L4–L5 spinal cord section was harvested for WB (Figure 4(a)) and IF (Figure 5(a)) analyses. The results showed that the expression of the KCC_2_ protein in rats in the LIFU^+^ group was upregulated compared with those in the LIFU^−^ group (*P* < 0.05). There was no difference between the normal and sham groups (*P* > 0.05; Figures 4(b) and 5(b)).

### 3.4. LIFU Stimulation Reduced the Expression of CaMKIV and p-CREB but Not of p-ERK1/2 in the L4–L5 Spinal Cord Section of PNI Rats

PNI activates the MAPK pathway and leads to high expression of CaMKIV, p-ERK, and p-CREB [41]. In this study, WB (Figures 4(c)–4(h)) showed that the expression of CaMKIV, p-ERK1/2, and p-CREB increased in the LIFU^−^ group. After 4 consecutive weeks of LIFU treatment, the expression of CaMKIV and p-CREB decreased compared with the LIFU^−^ group (*P* < 0.05; Figures 4(f) and 4(h)). IF also showed that the expression of p-CREB decreased after LIFU stimulation for 4 weeks compared with the LIFU^−^ group (*P* < 0.05; Figure 5(d)). Interestingly, there was no statistical difference in the p-ERK1/2 expression between the LIFU^−^ and LIFU^+^ groups, nor any significant difference in CaMKIV, p-ERK1/2, or p-CREB expression between the normal and sham groups (*P* > 0.05; Figures 4(d), 4(f), and 4(h)).

## 4. Discussion

Potassium chloride (K^+^-Cl^−^) cotransporter 2 (KCC_2_) is the only cationic chloride cotransporter expressed in mammalian neurons. It plays a prominent role in maintaining low [Cl^−^]*_i_*, which is necessary for the function of GABA_A_ and glycine receptors (GlyRs) and for mediating spinal cord inhibition [8, 10]. After intrathecal application of KCC_2_ inhibitor (2-[[(2S)-2-butyl-6,7-dichloro-2-cyclopentyl-1-oxo-3H-inden-5-yl]oxy], or DIOA), heat-evoked withdrawal latency and innocuous brush stimulation are significantly reduced [16, 42]. Our experimental data indicated that KCC_2_ was downregulated in the PNI group, and PWT_50_ was also lower in this group than in the normal and sham operation groups. All results showed that PNI led to downregulation of the KCC_2_ expression, which weakens GABA_A_/GlyR-mediated inhibition and then leads to NP [43]. All of the above changes are important factors contributing to the development and maintenance of NP. To further investigate the mechanism of NP, we found that enhancing the KCC_2_ function pharmacologically restored spinal cord inhibition and reduced allodynia [9]. In our study, pain behavior improved (Figure 2(b)), and the KCC_2_ expression was upregulated (Figure 4(b)) after 4 weeks of LIFU stimulation. Therefore, the expression of KCC_2_ in the spinal cord played an important role in the pathogenesis of NP, and upregulation of the KCC_2_ expression could potentially alleviate NP.

After PNI, the downregulation of KCC_2_ is closely related to activation of the BDNF–TrkB pathway and intracellular cascade reactions mediated by CaMKIV, p-CREB, and p-ERK [21–24]. Intrathecal application of a TrkB blocker significantly improves downregulation of the KCC_2_ expression on the membranes of spinal dorsal horn neurons induced by inflammatory pain [44]. In Kitayama's research, short interfering RNA (siRNA) was used to knock down zinc transporter-1 (ZnT-1), which led to inhibition of the BDNF–TrkB pathway, downregulation of p-CREB, upregulation of KCC_2_, and improvement of the withdrawal threshold [19]. After intrathecal injection of p-ERK blockers, chronic NP induced by oxaliplatin was also significantly alleviated in rats [45]. López-Alvarez and Li applied electroacupuncture to stimulate rats with chronic constriction injury (CCI) and found that it could effectively improve the KCC_2_ expression, the mechanical withdrawal threshold, and thermal withdrawal latency [18, 20]. Therefore, inhibition of the BDNF–TrkB pathway and cascade reactions mediated by CaMKIV, p-CREB, and p-ERK, as well as upregulation of the KCC_2_ expression, could effectively alleviate NP. In this study, we stimulated the spinal cord with LIFU and confirmed the efficacy of LIFU in treating NP. To our knowledge, this study was the first to use LIFU to stimulate the spinal cord in order to regulate NP.

Moreover, we found that CaMKIV and p-CREB were downregulated (Figures 4(f), 4(h), and 5(d)), and KCC_2_ upregulated (Figures 4(a) and 5(b)) after LIFU stimulation. Upregulation of the KCC_2_ expression can reduce neural [Cl^−^]_i_, increase the effect of GABA, and enhance the inhibitory effect of interneuron on the spinal cord, so that the pain threshold of sensory neurons in the spinal cord is reduced and the behavior of pathological pain is relieved [7, 46]. Therefore, we speculate that LIFU might alleviate pathological pain due to PNI by inhibiting CaMKIV and p-CREB expression and upregulating the KCC_2_ expression in neurons.

Interestingly, LIFU stimulation did not change the expression of p-ERK1/2 in the spinal cords of PNI rats (Figure 4(d)). While the exact underlying mechanism is unknown, there are several possible explanations. First, in NP rat models, the BDNF–TrkB pathway can activate CaMKIV by increasing the concentration of Ca^2+^ in neurons via the PLC*γ*–IP_3_ pathway, whereas p-ERK is activated through the TrkB–Ras pathway [22, 26]. Second, CaMKIV activation depends on the concentration of Ca^2+^ in neurons. The mechanical forces of LIFU can affect voltage-gated calcium and sodium channels (VGCCs, VGSCs) in the plasma membrane [35, 47], causing transient intracellular Ca^2+^ concentration changes in various cells [48]. This mechanism can be used in treatments such as mesenchymal stem cell (MSC) homing [36], neuromodulation in the brain [47], or immunotherapy with tumor US [49]. Therefore, we propose that LIFU might affect CaMKIV activation by interfering with the transient concentration of Ca^2+^ in neurons but without affecting the p-ERK1/2 expression. However, the specific mechanism of action remains unclear, requiring further research.

As a noninvasive neuromodulatory method, LIFU has many advantages such as higher spatial resolution, greater penetration depth, and no tissue damage [50]. As a nonthermal form of US, LIFU has litter thermal effect on local tissues. When peripheral focused US (pFUS; *F* = 1.15 MHz; peak negative pressure [PNP] = 4 MPa; DC = 5%) that is used to irradiate muscle and kidney tissue in vitro, the temperatures of these tissues increase by 1.1°C and 0.7°C, respectively [36]. In this study, we transected the spinal cord at L4–L5 and performed H&E staining to observe the safety of LIFU on the spinal cord. Our results showed no swelling, nuclear fragmentation of neurons, neutrophil infiltration, or bleeding (Figures 3(a) and 3(b)). Therefore, these results indicated that LIFU was a safe method for treating the spinal cord.

Overall, our study demonstrated that (i) LIFU stimulation of the spinal cord could effectively improve neuropathic pain behavior induced by peripheral nerve injury, which has potential value in the clinical treatment of NP; (ii) LIFU stimulation of the spinal cord might affect the expression of CaMKIV, CREB, and KCC_2_; and (iii) stimulation of the spinal cord with LIFU was safe.

## 5. Limitations

Our study had some limitations. First, we established only a short treatment period and did not evaluate the long-term efficacy of US therapy. Second, we selected only one time point at which to measure the expression of CaMKIV, p-CREB, p-ERK, and KCC_2_. Third, we found that LIFU could affect the expression of CaMKIV, p-CREB, and KCC_2_, but we failed to explore the specific mechanism by which it affected the expression of the above proteins. Thus, further experiments are needed.

## 6. Conclusions

We found that LIFU could effectively alleviate NP in rats with PNI by increasing the expression of KCC_2_ in the spinal dorsal corner. Moreover, LIFU upregulated the expression of KCC_2_, possibly by inhibiting activation of the CaMKIV–KCC_2_ pathway.

## Data Availability

The data used to support the findings of this study are available from the corresponding author upon request.

## Abbreviations

NP: Neuropathic pain
LIFU: Low-intensity focused ultrasound
PNI: Peripheral nerve injury
KCC_2_: Potassium chloride cotransporter 2
CaMKIV: Calcium/calmodulin-dependent protein kinase type IV
p-CREB: Phosphorylated cyclic adenosine monophosphate response element-binding protein
p-ERK1/2: Phosphorylated extracellular signal–regulated kinase 1/2
GABA: *γ*-Aminobutyric acid
BDNF: Brain-derived neurotrophic factor
TrkB: Tropomyosin receptor kinase B
CNS: Central nervous system
dps: Days postsurgery
PWT_50_: 50% paw withdrawal threshold.

## References

[1] Haanpää M., Attal N., Backonja M. (2011). NeuPSIG guidelines on neuropathic pain assessment. *Pain*.

[2] Finnerup N. B., Haroutounian S., Kamerman P. (2016). Neuropathic pain: an updated grading system for research and clinical practice. *Pain*.

[3] Bouhassira D., Attal N. (2016). Translational neuropathic pain research: a clinical perspective. *Neuroscience*.

[4] Grinsell D., Keating C. P. (2014). Peripheral nerve reconstruction after injury: a review of clinical and experimental therapies. *BioMed Research International*.

[5] van Hecke O., Austin S. K., Khan R. A., Smith B. H., Torrance N. (2014). Neuropathic pain in the general population: a systematic review of epidemiological studies. *Pain*.

[6] Colloca L., Ludman T., Bouhassira D. (2017). Neuropathic pain. *Nature Reviews Disease Primers*.

[7] Wright R., Newey S. E., Ilie A. (2017). Neuronal chloride regulation via KCC2 is modulated through a GABABReceptor protein complex. *The Journal of Neuroscience*.

[8] Tillman L., Zhang J. (2019). Crossing the Chloride Channel: The Current and Potential Therapeutic Value of the Neuronal K+-Cl- Cotransporter KCC2. *BioMed Research International*.

[9] Gagnon M., Bergeron M. J., Lavertu G. (2013). Chloride extrusion enhancers as novel therapeutics for neurological diseases. *Nature Medicine*.

[10] Kaila K., Price T. J., Payne J. A., Puskarjov M., Voipio J. (2014). Cation-chloride cotransporters in neuronal development, plasticity and disease. *Nature Reviews Neuroscience*.

[11] Costa E. (1998). From GABAARECEPTOR diversity emerges a unified vision of GABAergic inhibition. *Annual Review of Pharmacology and Toxicology*.

[12] Macdonald R. L., Olsen R. W. (1994). GABAAReceptor channels. *Annual Review of Neuroscience*.

[13] Duan B., Cheng L., Ma Q. (2018). Spinal circuits transmitting mechanical pain and itch. *Neuroscience Bulletin*.

[14] Todd A. J. (2015). Plasticity of inhibition in the spinal cord. *Handbook of Experimental Pharmacology*.

[15] Zhang J., Yu J., Kannampalli P. (2017). MicroRNA-mediated downregulation of potassium-chloride-cotransporter and vesicular *γ*-aminobutyric acid transporter expression in spinal cord contributes to neonatal cystitis-induced visceral pain in rats. *Pain*.

[16] Austin T. M., Delpire E. (2011). Inhibition of KCC2 in mouse spinal cord neurons leads to hypersensitivity to thermal stimulation. *Anesthesia and Analgesia*.

[17] Kitayama T. (2018). The role of K(+)-cl(-)-cotransporter-2 in neuropathic pain. *Neurochemical Research*.

[18] Lopez-Alvarez V. M., Cobianchi S., Navarro X. (2019). Chronic electrical stimulation reduces hyperalgesia and associated spinal changes induced by peripheral nerve injury. *Neuromodulation*.

[19] Kitayama T., Morita K., Motoyama N., Dohi T. (2016). Down-regulation of zinc transporter-1 in astrocytes induces neuropathic pain via the brain-derived neurotrophic factor - K^+^-Cl^−^ co-transporter-2 signaling pathway in the mouse spinal cord. *Neurochemistry International*.

[20] Li S. S., Tu W. Z., Jia C. Q. (2018). KCC2-GABAA pathway correlates with the analgesic effect of electro-acupuncture in CCI rats. *Molecular Medicine Reports*.

[21] Tsuda M. (2019). Microglia-mediated regulation of neuropathic pain: molecular and cellular mechanisms. *Biological & Pharmaceutical Bulletin*.

[22] Lee-Hotta S., Uchiyama Y., Kametaka S. (2019). Role of the BDNF-TrkB pathway in KCC2 regulation and rehabilitation following neuronal injury: a mini review. *Neurochemistry International*.

[23] Rivera C., Voipio J., Thomas-Crusells J. (2004). Mechanism of activity-dependent downregulation of the neuron-specific K-cl cotransporter KCC2. *The Journal of Neuroscience*.

[24] Coull J. A., Beggs S., Boudreau D. (2005). BDNF from microglia causes the shift in neuronal anion gradient underlying neuropathic pain. *Nature*.

[25] Obata K., Noguchi K. (2004). MAPK activation in nociceptive neurons and pain hypersensitivity. *Life Sciences*.

[26] Ma W., Quirion R. (2005). The ERK/MAPK pathway, as a target for the treatment of neuropathic pain. *Expert Opinion on Therapeutic Targets*.

[27] Woolf C. J. (2010). Overcoming obstacles to developing new analgesics. *Nature Medicine*.

[28] Kuffler D. P. (2020). Mechanisms for reducing neuropathic pain. *Molecular Neurobiology*.

[29] DiBonaventura M. D., Sadosky A., Concialdi K. (2017). The prevalence of probable neuropathic pain in the US: results from a multimodal general-population health survey. *Journal of Pain Research*.

[30] Caruso R., Ostuzzi G., Turrini G. (2019). Beyond pain: can antidepressants improve depressive symptoms and quality of life in patients with neuropathic pain? A systematic review and meta-analysis. *Pain*.

[31] Li X., Yang H., Yan J., Wang X., Yuan Y., Li X. (2019). Seizure control by low-intensity ultrasound in mice with temporal lobe epilepsy. *Epilepsy Research*.

[32] Eguchi K., Shindo T., Ito K. (2018). Whole-brain low-intensity pulsed ultrasound therapy markedly improves cognitive dysfunctions in mouse models of dementia - crucial roles of endothelial nitric oxide synthase. *Brain Stimulation*.

[33] Chen S. F., Su W. S., Wu C. H., Lan T. H., Yang F. Y. (2018). Transcranial ultrasound stimulation improves long-term functional outcomes and protects against brain damage in traumatic brain injury. *Molecular Neurobiology*.

[34] Zhang D., Li H., Sun J. (2019). Antidepressant-like effect of low-intensity Transcranial ultrasound stimulation. *IEEE Transactions on Biomedical Engineering*.

[35] Tyler W. J., Lani S. W., Hwang G. M. (2018). Ultrasonic modulation of neural circuit activity. *Current Opinion in Neurobiology*.

[36] Burks S. R., Lorsung R. M., Nagle M. E., Tu T. W., Frank J. A. (2019). Focused ultrasound activates voltage-gated calcium channels through depolarizing TRPC1 sodium currents in kidney and skeletal muscle. *Theranostics*.

[37] Kubanek J., Shukla P., Das A., Baccus S. A., Goodman M. B. (2018). Ultrasound elicits behavioral responses through mechanical effects on neurons and ion channels in a simple nervous system. *The Journal of Neuroscience*.

[38] King R. L., Brown J. R., Newsome W. T., Pauly K. B. (2013). Effective parameters for ultrasound-induced _in vivo_ neurostimulation. *Ultrasound in Medicine & Biology*.

[39] Decosterd I., Woolf C. J. (2000). Spared nerve injury: an animal model of persistent peripheral neuropathic pain. *Pain*.

[40] Chaplan S. R., Bach F. W., Pogrel J. W., Chung J. M., Yaksh T. L. (1994). Quantitative assessment of tactile allodynia in the rat paw. *Journal of Neuroscience Methods*.

[41] Xu X., Fu S., Shi X., Liu R. (2019). Microglial BDNF, PI3K, and p-ERK in the spinal cord are suppressed by pulsed radiofrequency on dorsal root ganglion to ease SNI-induced neuropathic pain in rats. *Pain Research and Management*.

[42] Mapplebeck J. C. S., Lorenzo L. E., Lee K. Y. (2019). Chloride dysregulation through downregulation of KCC2 mediates neuropathic pain in both sexes. *Cell Reports*.

[43] Prescott S. A. (2015). Synaptic inhibition and disinhibition in the spinal dorsal horn. *Progress in Molecular Biology and Translational Science*.

[44] Tsuruga K., Hashimoto T., Kato R. (2016). Plantar injection of formalin in rats reduces the expression of a potassium chroride cotransporter KCC2 in the spinal cord and a kinase inhibitor suppresses this reduction. *Biomedical Research*.

[45] Maruta T., Nemoto T., Hidaka K. (2019). Upregulation of ERK phosphorylation in rat dorsal root ganglion neurons contributes to oxaliplatin-induced chronic neuropathic pain. *PLoS One*.

[46] Coull J. A., Boudreau D., Bachand K. (2003). Trans-synaptic shift in anion gradient in spinal lamina I neurons as a mechanism of neuropathic pain. *Nature*.

[47] Tyler W. J., Tufail Y., Finsterwald M., Tauchmann M. L., Olson E. J., Majestic C. (2008). Remote excitation of neuronal circuits using low-intensity, low-frequency ultrasound. *PLoS One*.

[48] Suarez Castellanos I. M., Balteanu B., Singh T., Zderic V. (2016). Therapeutic modulation of calcium dynamics using ultrasound and other energy-based techniques. *IEEE Reviews in Biomedical Engineering*.

[49] Kim K. D., Bae S., Capece T. (2017). Targeted calcium influx boosts cytotoxic T lymphocyte function in the tumour microenvironment. *Nature Communications*.

[50] Landhuis E. (2017). Ultrasound for the brain. *Nature*.

